# A randomized, placebo-controlled laboratory study of the effects of D-cycloserine on sexual memory consolidation in women

**DOI:** 10.1007/s00213-020-05457-4

**Published:** 2020-01-27

**Authors:** S. Both, R. J. B. Van Veen, M. Brom, P. T. M. Weijenborg

**Affiliations:** Department of Psychosomatic Gynecology and Sexology, Leiden University Medical Center, Poortgebouw-Zuid, 4e etage, Rijnsburgerweg 10, 2333 AA Leiden, The Netherlands

**Keywords:** Memory consolidation, Learning, NMDA, Reward, Sexual arousal, Classical conditioning

## Abstract

**Rationale and objective:**

The aim of this study was to investigate the possible facilitating effect of the partial NMDA receptor agonist D-cycloserine (DCS) on memory consolidation of conditioned sexual responses and to examine the capability of DCS to reduce context-specificity of learning.

**Methods:**

In a randomized placebo-controlled double-blind trial, 50 healthy females were exposed to a differential conditioning procedure. Two pictures of a male abdomen were used as conditional stimuli (CSs), of which one (the CS+) was followed by the unconditional stimulus (US), a genital vibrotactile stimulus. After the conditioning session on day 1, participants received either 125 mg of DCS or a placebo. The effects of DCS on affect, sexual arousal and US expectancy in response to the CS+ and CS− were examined 24 h after the conditioning procedure.

**Results:**

A main effect of DCS was found on affect at the first test trials (*p* = 0.04, *η*_*p*_^*2*^ = 0.09), and a similar non-significant but trend level effect was found for sexual arousal (*p* = 0.06, *η*_*p*_^*2*^ = 0.07), which appeared to persist over a longer time (*p* = 0.07, *η*_*p*_^*2*^ = 0.08). Unexpectedly, ratings of positive affect and sexual arousal in response to *both* the CS+ and the CS− were higher in the DCS condition compared to the control condition, possibly indicating that DCS administration reduced stimulus specificity. Since the results did not show clear evidence for context learning, we were not able to test effects on context-specificity of learning.

**Conclusion:**

Although largely inconclusive, the results provide tentative support for a facilitating effect of DCS on affect and sexual arousal in response to stimuli that were presented in a sexual conditioning procedure, however, no conclusions can be drawn about effects of DCS on sexual reward learning, since the design and results do not lend themselves to unambiguous interpretation.

D-cycloserine (DCS) is known to influence N-methyl-D-aspartate (NMDA) receptors in the brain (Sheinin et al. 2001). The NMDA receptor is one of the receptors via which glutamate can alter a broad range of learning and memory processes by interacting with cortical and subcortical circuits (Fitzgerald et al. 2014). One of the learning and memory processes that can be altered is long-term potentiation, which is postulated to underlie new learning (Citri and Malenka 2008; Feldman 2009; Forsyth et al. 2015). Activation of NMDA receptors appears to be important for successful consolidation of new learning (Marin et al. 2015). DCS has been shown to enhance acquisition, consolidation, extinction and reconsolidation in several associative learning paradigms in rodents and humans (Botreau et al. 2006; Brom et al. 2015; Kalisch et al. 2009; Kuriyama et al. 2011; Ledgerwood et al. 2003, 2005; Myers and Carlezon Jr. 2012; Parnas et al. 2005; Ressler et al. 2004; Torregrossa et al. 2010). Furthermore, the results of studies using NMDA antagonists, as opposed to NMDA agonists, showed blocking effects on memory consolidation and reconsolidation (Alaghband and Marshall 2013; Feltenstein and See 2007; Milton et al. 2008).

DCS has been studied as an additive to cognitive behavioural therapy (CBT) in order to facilitate new learning. Systematic meta-analyses of studies in which anxiety patients were administered DCS or placebo and received exposure-based therapy showed support for a small effect of DCS in enhancing extinction learning (Mataix-Cols et al. 2017; Rodrigues et al. 2014). Besides extinction of aversive responses, DCS has also been found to enhance extinction of appetitive responses. Studies in rats showed enhanced extinction of conditioned drug-seeking behaviour in the DCS group compared to the control group and indicated resistance to reinstatement (Botreau et al. 2006; Paolone et al. 2009; Vengeliene et al. 2008). Also, in humans, DCS was found to facilitate extinction of appetitive responses. Studies in cigarette smokers or problematic drinkers receiving cue exposure therapy and either placebo or DCS, showed reduced physiological reactivity as well as reduced craving while being exposed to drug-associated cues in the DCS group compared to the placebo group (Kiefer et al. 2015; MacKillop et al. 2015; Otto et al. 2018; Santa Ana et al. 2009). The evidence is mixed, however, since there are also several studies with negative findings (Kamboj et al. 2012; Price et al. 2013; Prisciandaro et al. 2013; Santa Ana et al. 2015). Recently, our research group observed a facilitating effect of DCS on extinction learning of sexual responses (Brom et al. 2015). In this study, participants were sexually conditioned to a picture by using genital vibrostimulation as unconditioned stimulus. Afterwards, extinction learning took place and participants received either placebo or DCS. Increased extinction of genital and subjective sexual response in the DCS group compared to the control group was found. Interestingly, the effects in the DCS group where context-independent in contrast to the placebo group. The reduction in context specificity by DCS was found in other studies on extinction of appetitive stimuli as well. In these studies, DCS administration reduced the context specificity of the extinction of cocaine-associated cues in rats (Torregrossa et al. 2013; Torregrossa et al. 2010). Taken together, studies suggest that DCS facilitates consolidation of new learning, and reduces context specificity, and can be useful as pharmaceutical addition to associative learning-based psychological treatments.

So far, research on DCS as addition to psychological treatment mostly concerns treatment that is focused on extinction of fear, such as in anxiety disorders, or on extinction of maladaptive strong motivational responses to drug cues, such as in drug addiction. However, pharmacological enhancement of CBT for disorders characterized by maladaptive *low* motivation may also be an interesting option. CBT for maladaptive low motivation such as in anhedonia or low sexual interest strives to target deficits in appetitive responding, by enhancing the anticipation, consumption and learning of reward (Both, Laan, & Schultz 2010; Both et al. 2017b; Craske et al. 2016; ter Kuile et al. 2010). A recent study in rats showing a facilitating effect of a new NMDA agonist, Rapastinel, on positive emotional learning (Khan et al. 2018), and older studies showing enhancing effects of DCS on socio-sexual behaviour (McAllister 1994) and conditioned flavour-taste preference (Golden and Houpt 2007), indicate that DCS may be helpful in the facilitation of appetitive learning. Research in humans on the potential enhancing effect of DCS on appetitive learning is very scarce. In a study on the effect of DCS on complex reward-guided decision making, it was found that DCS shifted decision-making towards more optimal integration of reward probability and magnitude information, pointing to a facilitating effect of DCS on instrumental reward reversal learning (Scholl et al. 2014). However, studies in humans on the effect of DCS on simpler associative appetitive learning have to our knowledge not been done. In the present study, it was tested whether DCS can facilitate the acquisition of appetitive responses, more specifically sexual responses. Enhancement of the acquisition of appetitive sexual responses is of interest in the context of problems of low sexual interest and arousal, which are relatively common in women (Both et al. 2010; Kingsberg et al. 2015; Kingsberg and Rezaee 2013). Female sexual interest/arousal disorder (FSIAD; American Psychiatric Association 2013) is generally treated by CBT which appears to have a positive effect, however, FSIAD is also considered as difficult to treat, and strategies to enhance treatment are welcome (Both et al. 2010; Wincze and Carey 2001).

The present study took a first step in providing insight in the possibilities of using DCS in the treatment of problems related to low motivation, such as FSIAD. The first aim of this study was to investigate the possible enhancing effects of DCS on memory consolidation in a classical sexual conditioning paradigm. The second aim was to investigate the influence of DCS on context specificity of conditioned sexual responses. Healthy sexually functional women served as participants within a double-blind, placebo-controlled randomized design, and were sexually conditioned to a picture by using genital vibrostimulation as unconditional stimulus. Similar to previous studies in our lab, appetitive sexual conditioning effects were examined by assessing positive-negative affect, sexual arousal and US expectancy in response to this stimulus (Both et al. 2011; Both et al. 2008; Brom et al. 2014b). First, it was hypothesized that participants who are given 125 mg of DCS after the acquisition of conditioned sexual responses will show enhanced memory of the conditioned sexual response compared to participants given a placebo. This difference will be seen on sexual response measures (conditioned affect and sexual arousal) in a test phase 24 h after acquisition. Second, based on the previous finding by our research group (Brom et al. 2015), it was hypothesized that for participants in the DCS condition, the memory of the conditioned sexual response will also be enhanced outside of the acquisition context compared to the placebo group on the sexual response measures. This would indicate that DCS reduces context specificity of the conditioned sexual response.

## Method

### Participants

Fifty heterosexual women from the general population participated in the study and gave written consent before participation. Participants were pre-assessed by means of a questionnaire and telephonic interview to exclude those currently under any medication or treatment, those with past or present mental or neurological illness, kidney impairment, those with a medical illness or use of medication that could interfere with sexual response or DCS and allergy to antibiotics. Participants were tested individually by a trained female experimenter. The study was approved by the Ethical Committee of the Medical Centre. Participants were randomly allocated to the DCS or placebo condition (see Table 1 for descriptive statistics).

**Table 1:** Descriptive variables of participants

	Placebo (*n* = 24)		DCS (*n* = 26)		*p*
	Mean	SD	Mean	SD	
Age	23.08	4.65	23.27	2.75	0.197
Sexual functioning (FSFI)	28.36	4.44	27.02	7.29	0.135
Prior experience vibrostimulation	2.50	1.25	2.92	1.47	0.484

*FSFI*, Female Sexual Function Index (Ter Kuile et al., 2006). Item from the questionnaire on day 1: Prior experience with vibrostimulation (never (1), very often (5))

### Stimulus materials

Two identical pictures served as stimulus materials (CSs) and portrayed a male abdomen (wearing underwear), with the colour of the depicted underwear (blue or yellow) being the only difference (Brom et al. 2015). The CSs were shown for 9 s. Assignment of the pictures as CS+ and CS− was counterbalanced across participants and conditions.

### Genital vibrostimulation

The genital vibrostimulation (US) was administered by means of a small hands-off vibrator (2 cm diameter) (Brom et al. 2014a, b). The vibrator was placed on the clitoris using a Lycra panty. The participants were instructed to place the vibrator in such a way it was most sexually stimulating. On day 1, the vibrostimulation was provided only during the acquisition phase, 8 s following the start of the CS+ for 2 s. A reinforcement ratio of 80% was chosen (8 out of 10 CS+ presentations are followed by genital vibrostimulation) to increase reward prediction uncertainty (Schultz et al. 1997) in order to make conditioning somewhat more extinction resistant and increase the likelihood of recall of sexual reward memory on day 2. On day 2, reinstatement of the sexual memory in context A was facilitated by additionally presenting unpaired US of 2 s at the beginning of each context A block, thus again firmly associating context A with the US.

### Context manipulation

To investigate whether DCS can reduce context specificity of acquisition of reward-associated cues in humans, conditioning occurred in 2 different contexts in order to create a context-dependent memory. Contexts were manipulated by illuminating the experimental room in either a pink or yellow light (Brom et al. 2014a, b). Lighting was supplied by a frame with six fluorescent tubes of 36 W (two pink and four yellow tubes). The experimenter controlled the lighting from an adjacent room. The colours of the lighting that served as contexts A and B were randomly counterbalanced across participants.

### Subjective ratings

To study sexual incentive learning, positive-negative affect, sexual arousal and sexual reward expectancy elicited by the CSs were examined. Appetitive and aversive conditioning can result in a change in the strength of sexual motivation for a stimulus, but also in a change in the more general hedonic value (like-dislike) of a stimulus reflected in feelings of positive-negative affect (Berridge & Kringelbach 2008; Domjan & Gutierrez 2019). Although, in general, stronger feelings of sexual arousal in response to a stimulus will go together with more liking of this stimulus, studies on sexual responding have shown that sexual arousal and positive-negative affect can be relatively independent (Brom et al. 2015; Janssen 2007), therefore, both affect and sexual arousal were assessed. US expectancy was assessed to examine whether repeated pairing of the CS+ with the US during the learning phase, resulted in stronger expectation of the US at presentation of the CS+. Ratings of affective value, sexual arousal and US expectancy were collected during the preconditioning and conditioning phase on day 1 and during all context blocks on day 2. Participants were asked to rate, after each CS presentation, the affective value of the CSs by answering the question ‘What kind of feeling does this picture evoke in you?’ The question could be answered on a 7-point Likert scale on a keyboard that varied from very negative to very positive. Then, subjective sexual arousal was rated by answering the question ‘How sexually arousing is this picture to you?’ The question could be answered on a 7-point scale that varied from not sexually arousing at all to very sexually arousing. Then, participants were required to rate the expectancy of a vibration following the presentation of each CS on a 7-point scale by answering the question ‘To what extent did you expect a vibration after this picture?’ The scale consisted of 7 points labelled from ‘certainly no vibration’ through ‘certainly a vibration’. The questions were presented at the monitor 1 s following the end of picture presentation. The time the question was shown was paced by the participant’s response; the time to respond was maximally 11 s. When the participant answered the first question, the next question was presented after 15 s.

### Drugs

DCS (King Pharmaceuticals, Leicester, UK) was orally administered as 1 capsule of 125 mg. Optimal dosing for DCS has not been established in experimental human studies (Kalisch et al. 2009; Myers and Carlezon Jr. 2012). Clinical studies suggest only moderate doses (50–125 mg) DCS facilitate NMDA receptor-dependent forms of synaptic plasticity as well as learning and memory (Rouaud and Billard 2003). DCS plasma concentrations peak within 2 h in sober subjects (van Berckel et al. 1998). Therefore, subjects were asked not to eat 2 h preceding the experiment, in order to facilitate DCS absorption and to assure high DCS plasma levels during the theoretical critical time window for NMDA-dependent memory consolidation of 1–2 h post-learning (van Berckel et al. 1998; Zhu et al. 2001). Subjects were asked to refrain from alcohol and other drugs on the evening before and during the experimental days. Capsules with microcrystalline cellulose served as placebo.

### Design

**Fig. 1:**
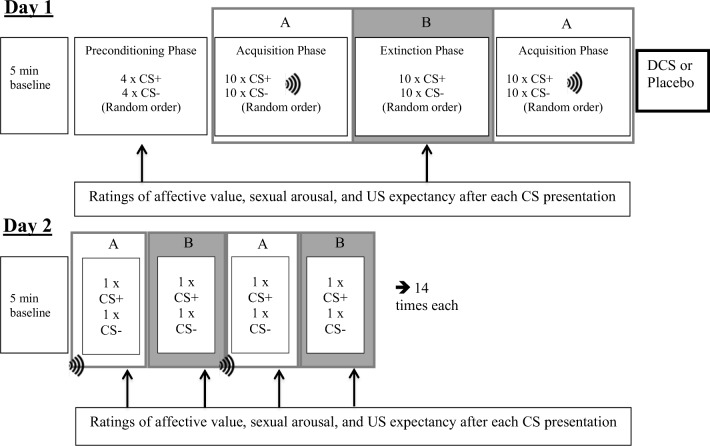
Schematic overview of the experimental procedure. During the baseline period, participants watched a neutral movie. The A blocks represent the acquisition context and the B blocks the extinction context.  = vibration

The design consisted of acquisition in context A and extinction in context B, see Fig. 1. The corresponding context was already present at the beginning of each block 8 s before CS presentation started. In the acquisition phase in context A, the CS+ and CS− were presented 10 times each and 8 out of 10 CS+ presentations were followed by the US. The extinction phase in context B consisted of 10 unreinforced CSs presentations. There were two random orders for each phase, with the restriction of only two successive presentations of each CS. There was no interval between the preconditioning, acquisition and extinction phases. During the whole procedure, inter-trial intervals (ITIs) were 20, 25 or 30 s. The order of the length of the ITI was random, with the restriction of only two successive lengths. To ascertain retention of sexual acquisition memories on day 2, conditioning was repeated in a further acquisition phase (A2) in its corresponding light context. Subjects received either DCS or placebo directly after the experiment on day 1 in a randomized, double-blind, between-subject fashion. Testing for the effects of DCS on CS-evoked conditioned responses (CRs) in both the acquisition (A) and the extinction context (B) took place 24 h later on day 2. Each context (A and B) was presented 14 times each, in alternating order (ABAB…) and in each context 1 CS+ and 1 CS− was presented. At the beginning of context A, subjects received an unpaired US of 2 s (i.e. not paired with the CS+ or CS−). Drug effects on consolidation were assessed by comparing the recall of sexual acquisition memories between the DCS and the placebo groups. Ratings of affective value, subjective sexual arousal and US expectancy were obtained after each CS-presentation in the preconditioning and extinction phase on day 1, and after each CS-presentation on day 2. On day 1, 40 min after drug intake, participants filled in an adverse symptoms checklist, for physical symptoms like dizziness, nausea and headache on a 4-point Likert scale (rated from 1 not present, 2 mild, 3 moderately severe, 4 extremely severe). Sixty minutes after drug intake, participants were allowed to leave the department.

### Data reduction, scoring and analysis

For the data collected at day 1, conditioning effects were tested comparing the response to the CS+ and CS− at the first trial of the extinction phase with mixed factor univariate analysis of variance procedures (general linear model in SPSS) with Stimulus as within-subject factor, and Condition (DCS or placebo) as between-subject factor. In addition, to test changes from pre-conditioning to the first extinction trial, a mixed factor ANOVA was performed with Stimulus and Trial as within-subject factors and Condition (DCS or placebo) as between-subject factor. Extinction in context B was tested comparing the response to the CS+ and CS− at the last trial of the extinction phase. In addition, to test changes from the first extinction trial to the last extinction trial, a mixed factor ANOVA was performed with Stimulus and Trial as within-subject factors and Condition (DCS or placebo) as between-subject factor.

For the data collected at day 2, effects were tested with mixed factor univariate analysis of variance procedures (general linear model in SPSS) with Stimulus, Context and Trial as within-subject factors and Condition (DCS or placebo) as between-subject factor. The initial effect was analysed on the first trials (one time CS+ and one time CS−) of each context, and in order to examine whether the effects are persistent over time, a second analyses was performed on the complete set of trials. The Greenhouse–Geisser correction was applied to adjust for violation of the sphericity assumption in testing repeated measures effects. All tests are two-tailed with an alpha of 0.05, and effect sizes are reported as proportion of partial variance (*η*_*p*_^*2*^), with a *η*_*p*_^*2*^ of 0.01, 0.06 and 0.14 indicating a respectively small, medium and large effect. Due to missing data in the subjective measures for day 2, for the persistence effect over trials, 21 participants from the placebo and 22 from the DCS condition were included for affect, 21 participants from the placebo and 20 from the DCS condition for sexual arousal, and 21 participants in both conditions for expectancy. With a chosen *p* value of 0.05, a power of 80% and an effect size of 0.5, a minimal number of 26 subjects was needed for within-subject effects (Cohen 1988). Recent conditioning studies (Brom et al. 2014a, b; Brom et al. 2015) demonstrated that 30 subjects within each condition are sufficient to observe between-subject effects. In addition, studies on the effects of DCS on extinction (Kalisch et al. 2009; Price et al. 2013; Santa Ana et al. 2009) were able to detect between-subject effects making use of 5–16 participants per condition. Unfortunately, with the inclusion of 50 participants and loss of data due to missing values, the study should be considered underpowered.

## Results

### Efficiency of blinding

Participants were asked 50 min after ingestion of the drugs on day 1 whether they thought they had received drug or placebo. In the DCS condition, 31% correctly thought they had received DCS, and in the placebo condition, 58% correctly thought they had received placebo. Within the DCS condition, participants guessed correctly below the chance level of 0.5 (*p* = 0.048), and within the placebo condition, participants did not guess correctly above or below chance level (*p* = 0.288), indicating that blinding was adequate. Most participants reported no side effects. Among the 23 participants (placebo *n* = 12; DCS *n* = 11) who reported side effects, the most commonly reported ones were lack of energy, sleepiness and headache.

### Day 1: Sexual conditioning

#### Preconditioning phase

Measures of the preconditioning phase were used to verify equal responses towards the CS+ and CS− and equal responses between conditions. On all of the measures (affect, sexual arousal and US expectancy) no differential responding to the CS+ and the CS− was found (all *p*s > 0.05). Herein, there was no difference between the placebo and the DCS condition (all *p*s > 0.05). For all of the measures, there were no significant Stimulus × Condition interactions (all *p*s > 0.05) (Table 2).

**Table 2:** Ratings of affect, sexual arousal and US expectancy following the CS+ and CS− on day 1 during the preconditioning phase and at the first and last extinction trial

	Placebo			DCS			Stimulus effect			Group effect			Stimulus X group effect		
	Mean (SD)	95% CI	*N*	Mean (SD)	95% CI	*N*	*F*	*p*	*η*_*p*_^*2*^	*F*	*p*	*η*_*p*_^*2*^	*F*	*p*	*η*_*p*_^*2*^
Pre-conditioning															
Affect															
CS+	4.36 (0.60)	4.18, 4.67	24	4.53 (0.60)	4.28, 4.78	26	1.26	0.262	0.03	2.29	0.145	0.05	0.25	0.620	0.01
CS−	4.42 (0.59)	4.22, 4.78		4.62 (0.78)	4.33, 4.89										
Sexual arousal															
CS+	3.66 (1.13)	3.32, 4.20	24	3.79 (1.09)	3.35, 4.23	26	3.64	0.062	0.07	0.36	0.553	0.01	0.37	0.548	0.01
CS−	3.77 (1.10)	3.41, 4.33		4.01 (1.23)	3.55, 4.47										
US expectancy															
CS+	3.13 (1.05)	2.72, 3.68	24	3.22 (1.36)	2.74, 3.70	26	1.32	0.256	0.03	0.33	0.570	0.01	1.56	0.218	0.03
CS−	3.17 (1.06)	2.72, 3.96		3.48 (1.40)	2.20, 3.97										
First extinction trial															
Affect															
CS+	4.71 (0.99)	4.35, 5.11	24	4.92 (0.91)	4.54, 5.31	25	5.01	0.029	0.10	2.77	0.103	0.06	1.18	0.282	0.09
CS−	4.25 (0.74)	4.01, 4.69		4.76 (0.93)	4.41, 5.12										
Sexual arousal															
CS+	4.29 (1.60)	3.82, 4.95	24	4.32 (1.25)	3.75, 4.89	25	1.27	0.266	0.03	0.09	0.765	0.00	0.08	0.780	0.00
CS−	3.96 (1.33)	3.52, 4.56		4.12 (1.33)	3.59, 4.65										
US expectancy															
CS+	4.26 (1.91)	3.52, 4.96	23	4.00 (1.76)	3.28, 4.72	25	9.49	0.003	0.17	0.06	0.811	0.00	0.22	0.642	0.01
CS−	2.96 (1.89)	2.40, 3.84		3.04 (1.65)	2.32, 3.76										
Last extinction trial															
Affect															
CS+	4.46 (1.14)	3.12, 4.05	24	3.54 (1.32)	3.02, 4.06	24	0.00	1.00	0.00	0.06	0.799	0.00	0.00	1.00	0.00
CS−	3.46 (1.06)	3.11, 4.10		3.54 (1.25)	3.05, 4.03										
Sexual Arousal															
CS+	2.46 (1.47)	1.97, 3.10	24	2.79 (1.41)	2.20, 3.38	24	0.07	0.784	0.00	1.61	0.210	0.03	1.21	0.277	0.03
CS−	2.25 (1.42)	1.77, 2.93		2.92 (1.53)	2.31, 3.52										
US expectancy															
CS+	1.67 (1.20)	1.18, 2.18	24	1.67 (1.2)	1.16, 2.18	24	0.02	0.903	0.00	0.55	0.462	0.01	1.79	0.186	0.04
CS−	1.46 (0.93)	1.00, 1.96		1.92 (1.41)	1.43, 2.40										

Ratings represent the following: extremely unpleasant (1) to extremely pleasant (7) for affective value, not sexually arousing at all (1) to very strongly sexually arousing (7) for sexual arousal and certainly no vibration (1) to certainly a vibration (7) for US expectancy

#### Conditioning effects

Measures on the first extinction trial were analysed to verify the conditioning effect (Table 2). Measures of affect and US expectancy showed conditioned responding, with higher affect and expectancy ratings in response to the CS+ compared to the CS− (*p*s < 0.05). Although the mean sexual arousal score on the CS+ was higher than the mean score on the CS−, unexpectedly, there was no significant stimulus effect on sexual arousal. No differences between the conditions were found for affect, sexual arousal and US expectancy (all *p*s > 0.10), and there were no significant Stimulus × Condition interactions (all *p*s > 0.05) (Table 2). Also, the Stimulus × Trial × Condition mixed ANOVA revealed a significant Stimulus × Trial interaction for affect, *F*(1,46) = 9.02, *p* = 0.004, *η*_*p*_^*2*^ = 0.16; sexual arousal, *F*(1,46) = 4.69, *p* = 0.035, *η*_*p*_^*2*^ = 0.09; and US expectancy, *F*(1,46) = 14.12, *p* < 0.001, *η*_*p*_^*2*^ = 0.24, indicating that from pre-conditioning to the first extinction trial, affect, sexual arousal and US expectancy in response to the CS+ showed a stronger increase relative to the response to the CS−, indicating a conditioning effect. There were no significant interactions with Condition.

Measures on the last extinction trial were analysed to verify extinction of conditioned responses in the extinction context B (Table 2). Measures of affect and US expectancy did no longer show conditioned responding, with no significant difference in affect and expectancy ratings in response to the CS+ compared to the CS− (*p*s > 0.10). Also, for sexual arousal, there was no significant difference in score on the CS+ compared to the CS− at the last extinction trial. No differences between the Conditions were found for affect, sexual arousal and US expectancy (all *p*s > 0.10), and there were no significant Stimulus × Condition interactions (all *p*s > 0.10) (Table 2). Also, the Stimulus × Trial × Condition mixed ANOVA revealed no significant effects for sexual arousal, but a significant Stimulus × Trial interaction for affect, *F*(1,46) = 5.67, *p* = 0.022, *η*_*p*_^*2*^ = 0.11, and US expectancy, *F*(1,46) = 9.03, *p* = 0.004, *η*_*p*_^*2*^ = 0.17, indicating that from the first to the last extinction trial, affect and US expectancy in response to the CS+ showed extinction. There were no significant interactions with Condition.

### Day 2: Recall of sexual memory

#### Affect

**Fig. 2:**
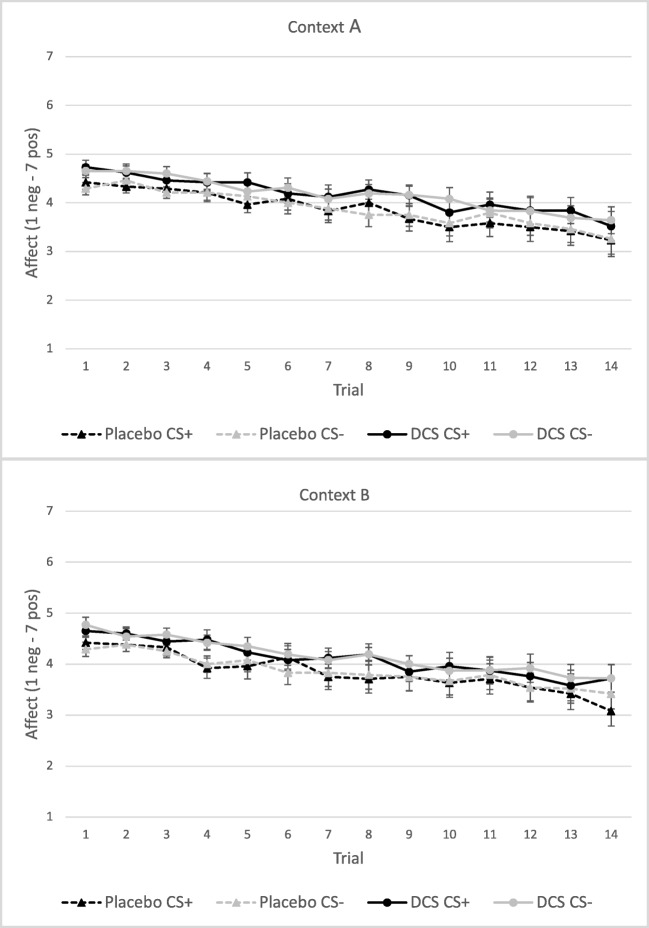
Mean affect ratings (± SEM) on day 2 following CS+ and CS− for the placebo and DCS condition per context, context A represents the acquisition context and context B the extinction context

For affect on the first trials, no main effect of Stimulus, main effect of Context, Stimulus × Condition interaction or Stimulus × Context × Condition interaction was found (all *p*s > 0.3). However, a main effect for Condition was observed, *F*(1,48) = 4.53, *p* = 0.038, *η*_*p*_^*2*^ = 0.09, with participants in the DCS condition rating higher than participants in the placebo condition (Fig. 2). Participants receiving DCS reported more positive affect towards both stimuli than participants receiving placebo and that this was the case regardless of the context. On the total amount of trials, no main effect of Stimulus, Context or Condition was found, and also, no interaction effect of Stimulus × Condition, or Stimulus × Context × Condition (all *p*s > 0.10), indicating that the enhancing effect of DCS on affect did not persist throughout the entire test period on day 2.

#### Sexual arousal

**Fig. 3:**
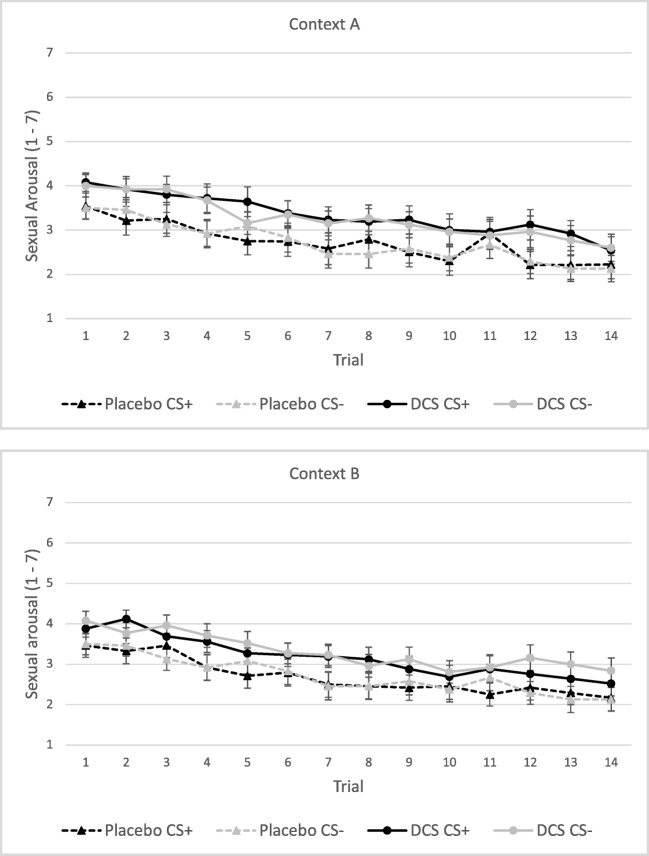
Mean sexual arousal ratings (± SEM) on day 2 following CS+ and CS− for the placebo and DCS condition per context, context A represents the acquisition context and context B the extinction context

On the first trials, no main effect of Stimulus or Context on sexual arousal was found. Furthermore, no interaction effect of Stimulus × Condition or Stimulus × Context × Condition was observed (all *p*s > 0.10). The main effect of Condition showed a trend towards significance, *F*(1,48) = 3.71, *p* = 0.060, *η*_*p*_^*2*^ = 0.07, with the DCS group scoring higher than the placebo group (Fig. 3). Participants in the DCS condition had a higher subjective sexual arousal following both stimuli compared to the participants in the placebo condition and this was regardless of the context.

On the total amount of trials, no main effect for Stimulus was found (*p* > 0.10). The main effect of Context showed a trend towards significance, *F*(1,39) = 3.41, *p* = 0.059, *η*_*p*_^*2*^ = 0.09, with higher sexual arousal ratings in the acquisition context. Also, the main effect of Condition showed a trend towards significance, *F*(1,39) = 3.41, *p* = 0.072, *η*_*p*_^*2*^ = 0.08, with the DCS group scoring higher than the placebo group (Fig. 3). Furthermore, the interaction of Stimulus × Context × Condition showed a trend towards significance *F*(1,39) = 3.58, *p* = 0.066, *η*_*p*_^*2*^ = 0.08. There were no other significant interaction effects, or interaction effects showing a trend towards significance (all *p*s > 0.12). Closer analysis of the trend level Stimulus × Context × Condition interaction, with analysis for the placebo and DCS condition separately, showed a significant Stimulus × Context interaction in the DCS condition, *F*(1,19) = 5.17, *p* = 0.036, *η*_*p*_^*2*^ = 0.23, but no significant Stimulus × Context interaction in the placebo condition (*p* > 0.7). However, further analyses in the DCS condition for both contexts separately revealed no significant effect of Stimulus for context A or context B (*p*s > 0.20), indicating that context A nor context B showed significant differences in ratings of sexual arousal in response to the CS+ and CS−. Taken together, these results carefully suggest that during the repeated test trials, the DCS group reported higher subjective sexual arousal in response to both the CS+ and CS− compared to the placebo group.

#### US expectancy

**Fig. 4:**
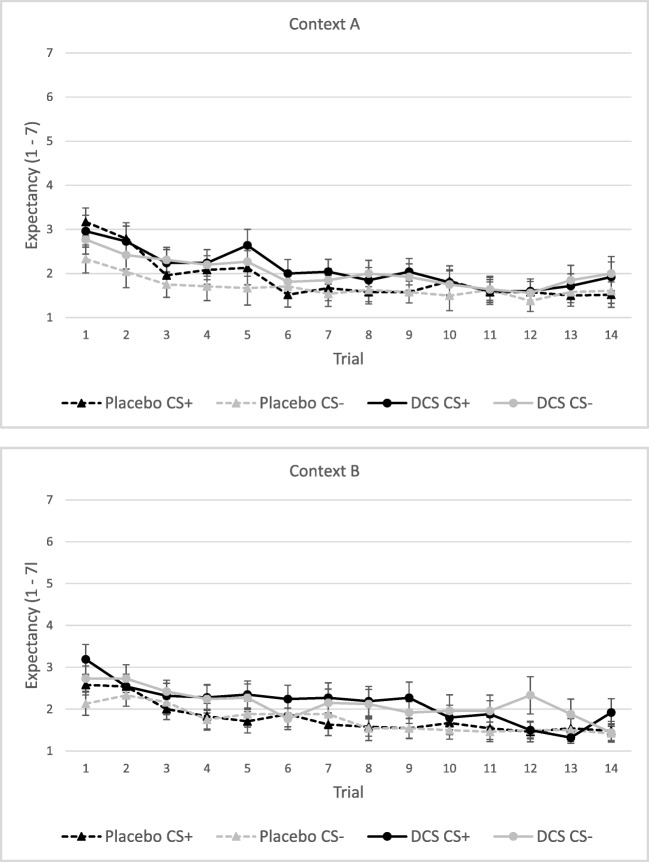
Mean US expectancy ratings (± SEM) at day 2 following CS+ and CS− for the placebo and DCS condition per context, context A represents the acquisition context and context B the extinction context

On the first trials, there was a significant main effect of Stimulus, *F*(1,48) = 6.94, *p* = 0.011, *η*_*p*_^*2*^ = 0.13, with US expectancy for the CS+ being rated higher than for the CS− (Fig. 4). No main effect for Condition or Context was found, neither was there an interaction effect of Stimulus × Condition or Stimulus × Context × Condition (all *p*s > 0.20).

On the total amount of trials, there was a significant main effect of Stimulus, *F*(1,40) = 4.60, *p* = 0.038, *η*_*p*_^*2*^ = 0.103, with US expectancy for the CS+ being scored higher than for the CS− (Fig. 4). No main effect of Condition or Context was found. Also, no interaction effect of Stimulus × Condition, or Stimulus × Context × Condition was observed (all *p*s > 0.10).

## Discussion

The aim of this study was to examine the possible enhancing effects of DCS on memory consolidation of conditioned sexual response. Furthermore, we aimed to investigate whether DCS could reduce context specificity of conditioned responding. It was hypothesized that participants in the DCS condition would show enhanced positive affect and sexual arousal following the CS+ compared to participants in the placebo condition in the test phase 24 h after the acquisition phase and that the context specificity of the learned sexual response would be reduced in the DCS condition compared to the placebo condition. For this aim, using a differential conditioning procedure, participants learned at day 1 that the CS+ was followed by a sexually rewarding US in the acquisition context A, and not in the extinction context B, while the CS− was never paired with the US.

First, the findings regarding the effects of the conditioning procedure at day 1 show that the acquisition procedure in context A resulted in significantly stronger US expectancy and feelings of positive affect in response to the CS+ compared to the CS− and that these responses extinguished as intended in context B. Unfortunately, sexual arousal ratings—although higher in response to the CS+ following the acquisition procedure—did not show significant conditioned responding. Based on the data of day 1, we conclude that for US expectancy and affect, we observed learning effects as intended, but conditioning of feelings of sexual arousal appeared to be less successful.

The results of the test phase, at day 2, do not show a facilitating effect of DCS on conditioned responses. Compared to participants in the placebo condition, participants in the DCS condition did not report significantly stronger feelings of positive affect or sexual arousal at day 2 in response to the CS+. Unexpectedly, positive affect and sexual arousal in response to both the CS+ and the CS− appeared to be higher in the DCS condition. This difference between conditions was significant for the affect ratings, and reached a non-significant trend level for the sexual arousal ratings. Only for the sexual arousal ratings, the non-significant trend level effect of DCS appeared to be persistent throughout the entire test period on day 2, indicating that the DCS effect on sexual arousal was more long-lasting than the effect on positive affect. It should be acknowledged, however, that the observed effects in the present study concerned non-significant trend level effects meaning that the conclusions drawn from these results should be interpreted with care.

For US expectancy ratings at day 2, we observed a main effect of Stimulus, but no main or interaction effects of Context or Condition. Thus, at day 2, regardless of Context or Condition, expectancy of the sexually rewarding US was higher following the CS+ compared to the CS−. On the complete set of trials, the Stimulus effect reached a trend level significance, carefully suggesting that this effect was persistent throughout the complete test period. These results show that the conditioning procedure of day 1 resulted in a stronger expectation of the US at presentation of the CS+ at day 2, but not predominantly in the acquisition context. Since context conditioning appeared not to be successful, our data do unfortunately not allow conclusions regarding a possible effect of DCS on context-specific learning. However, the observation that for US expectancy, different from affect and sexual arousal, there was no main effect of DCS, is in line with the results of the previous study in our lab showing effects of DCS on sexual arousal and affect but not on expectancy ratings (Brom et al. 2015). The lack of effect of DCS on expectancy could be explained by the level of learning and the brain structures involved as previously suggested (Brom et al. 2015). It is suggested, based on findings of animal and human studies, that DCS is more influential on lower-order rather than higher-order learning (Grillon 2009). Different brain structures are involved in these two orders of learning (Carter et al. 2006), and expectancy could be processed at a predominantly conscious level and be related to anticipation, which is associated with rather higher-order learning than lower-order learning. Therefore, expectancy can possibly remain relatively unaffected by DCS.

The observed effect of DCS on affect and sexual arousal in response to both the CS+ and the CS− was unexpected. Compared to the placebo group, participants receiving DCS following the conditioning procedure reported more positive affect and tended to show more sexual arousal towards both the CS+ and the CS− at day 2. This is of interest since it may possibly suggest that DCS can facilitate generalization of the learning to other stimuli than the CS+ which was initially associated with the US. Generalization of learning effects after administration of DCS was found in previous studies (Byrne et al. 2015; Ledgerwood et al. 2005; Vanvossen et al. 2017). In the study of Ledgerwood et al. (2005), rats were exposed to a fear-conditioning paradigm, with light or a tone as CS and a white-noise burst as US. Afterwards, the rats received extinction training (exposure) to one of the CSs. In the test phase, it appeared that DCS facilitated extinction of learned fear to the extinguished CS and that the extinction effect generalized to the non-extinguished CS, when DCS-treated rats were compared to placebo controls. Byrne et al. (2015) examined whether DCS enhanced generalization of fear extinction learning across different stimuli and contexts among children with specific phobias (dog and spider phobia). In this double-blind, placebo-controlled, randomized controlled trial, participants received 50 mg of DCS or placebo before a prolonged exposure session to the feared stimulus. One week later, fear response towards a different stimulus was examined in the treatment context and an alternate context. It was found that there was no difference between conditions with the new stimulus in the treatment context, in contrast to the alternate context, where participants in the DCS condition showed less avoidance and less fear towards the new stimulus compared to participants in the placebo condition. In addition, Vanvossen et al. (2017) observed that in rats, activation of prelimbic cortex NMDA receptors after acquisition of a contextual fear memory resulted in enhanced fear expression to another context, indicating generalized fear expression through enhanced fear memory consolidation. Ledgerwood et al. (2005) state that their results may point to DCS reducing stimulus specificity, since extinction training with one CS rarely results in a loss in responding to a different CS previously paired with the same US. Reduction of CS specificity by DCS administration may also explain our findings, especially since our CSs did not differ from each other to a great extent.

However, it should be noted that in the present study, differential conditioning was used as an index for the effect of DCS on learning, and at this stage, it is impossible to know whether the observed enhanced responding to both the CS+ and the CS− in the DCS condition is the result of the drug-enhancing memory and generalizability or the result of a direct non-task-related effect that would also be found in the absence of the conditioning procedure. It is possible that the stronger positive affect and sexual arousal towards the two stimuli at day 2 is an effect of the appetitive conditioning procedure at day 1, and it is this appetitive learning that DCS enhanced, but it is also possible that DCS just had a direct effect on affect. In the context of this question, it is relevant that we did not observe effects of drug on mood in the assessment of side effects. However, sexual arousal and affect were not measured prior to the recall task, so we can not preclude baseline differences in sexual arousal and affect prior to recall, and therefore we cannot draw conclusions regarding effects of DCS on learning. In order to test whether DCS enhances appetitive learning and reduces stimulus specificity, further research is necessary with the assessment of baseline responses prior to recall and inclusion of ‘new’ perceptual similar stimuli next to the CSs.

Another limitation of this study involves the lack of assessment of physiological sexual arousals. Assessment of both subjective and physiological sexual arousal would give a more complete picture of effects on sexual appetitive responding. Besides this, the length of the study could be considered as a limitation. Especially on the test day (day 2), the experimental session might have been experienced as long. The length was defined by the opportunity to see whether the effect lasted over time and the inter-trial intervals of 20 to 25 s to ensure that the arousal levels return to baseline in between trials. Possibly, the arousal level on the later trials might have decreased due to boredom. However, exit interview reports showed that the majority of participants were able to keep their eyes at the screen presenting the stimuli instead of being distracted. Another limitation is that the extinction procedure in context B on the first day might have influenced the conditioning process. Due to the extinction procedure in context B, which took place between the two acquisition phases in context A, and showed the CSs without the US, the acquisition effects might have been less effective. We included this extinction procedure to verify conditioning effects following the acquisition procedure at the first extinction trial and to enable examination of the effects of DCS on context learning. However, we should note, that with hindsight, this experimental design is less optimal, since an alternative explanation for the results may be that DCS interferes with the consolidation of extinction learning rather than facilitating acquisition. To test context generalization, in future studies, responses to the CSs can be better examined in a novel context at recall. Furthermore, it should be noted that since we presented the US at the start of the recall test in the acquisition context, actual reinstatement was tested rather than pure renewal.

Despite these limitations, our study contributes to research on the effects of DCS on (sexual) appetitive learning (Brom et al. 2015) and may contribute to the insights on the effectivity of DCS in addition to psychological treatments. As aforementioned, an enhancing effect of DCS has been shown in extinction learning of fear and anxiety in rats and humans (e.g. (Rodrigues et al. 2014) and in extinction learning of appetitive responses, although the findings from different studies in the appetitive domain are mixed (e.g. (Santa Ana et al. 2009). Although, as noted before, our results should be considered as inconclusive due to several limitations precluding inferences about learning effects, they may tentatively indicate that besides extinction learning, DCS may also facilitate memory consolidation of new positive sexual associations. And this can be relevant for treatment of low sexual interest and arousal disorder, but also for treatment for maladaptive low motivation such as in anhedonia which strive to target deficits in appetitive responding, by enhancing the anticipation, consumption and learning of reward (Both 2017; Both et al. 2010; Craske et al. 2016). However, to determine the relevance for treatment of disorders such as low sexual interest and arousal, of course, it should first be examined whether conditioning and effects of DCS in a sexually functional population can be extrapolated to populations with sexual dysfunction (Both et al. 2017a; Brom et al. 2014a).

Further study is highly recommendable since the sample size from the power calculation was not met which could lead to underpowered results and the experimental design had several limitations. Follow-up studies including a neutral context (ABC-design), assessment of baseline responses prior to recall and ‘new’ perceptual similar stimuli next to the CSs will lead to more insight in the possible effect of DCS on stimulus and context generalization. Also, with regard to clinical relevance, it would be interesting to see in future studies if the effects of DCS continue to exist over a longer time than 24 h. Furthermore, in future research, it is necessary to investigate optimal doses of DCS for memory consolidation enhancement. Doses used in previous studies were varying from 50 to 500 mg and an optimal dosage is not yet established (Rodrigues et al. 2014).

To conclude, to our knowledge, this is the first study exploring the possible facilitating effect of DCS on appetitive sexual responding by enhancing learning of sexual reward. Although largely inconclusive, the results provide tentative support for a facilitating effect of DCS on sexual arousal and affect, and may possibly point to DCS facilitating stimulus generalization. However, no conclusions can be drawn about effects of DCS on sexual reward learning, since the design and results do not lend themselves to unambiguous interpretation.
